# Role of an SNP in Alternative Splicing of Bovine *NCF4* and Mastitis Susceptibility

**DOI:** 10.1371/journal.pone.0143705

**Published:** 2015-11-24

**Authors:** Zhihua Ju, Changfa Wang, Xiuge Wang, Chunhong Yang, Yan Sun, Qiang Jiang, Fei Wang, Mengjiao Li, Jifeng Zhong, Jinming Huang

**Affiliations:** Dairy Cattle Research Center, Shandong Academy of Agricultural Sciences, Jinan, Shandong, 250131, China; International Centre for Genetic Engineering and Biotechnology, ITALY

## Abstract

Neutrophil cytosolic factor 4 (NCF4) is component of the nicotinamide dinucleotide phosphate oxidase complex, a key factor in biochemical pathways and innate immune responses. In this study, splice variants and functional single-nucleotide polymorphism (SNP) of *NCF4* were identified to determine the variability and association of the gene with susceptibility to bovine mastitis characterized by inflammation. A novel splice variant, designated as *NCF4*-*TV* and characterized by the retention of a 48 bp sequence in intron 9, was detected in the mammary gland tissues of infected cows. The expression of the *NCF4-reference* main transcript in the mastitic mammary tissues was higher than that in normal tissues. A novel SNP, g.18174 A>G, was also found in the retained 48 bp region of intron 9. To determine whether *NCF4*-*TV* could be due to the g.18174 A>G mutation, we constructed two mini-gene expression vectors with the wild-type or mutant *NCF4* g.18174 A>G fragment. The vectors were then transiently transfected into 293T cells, and alternative splicing of *NCF4* was analyzed by reverse transcription-PCR and sequencing. Mini-gene splicing assay demonstrated that the aberrantly spliced *NCF4*-*TV* with 48 bp retained fragment in intron 9 could be due to g.18174 A>G, which was associated with milk somatic count score and increased risk of mastitis infection in cows. *NCF4* expression was also regulated by alternative splicing. This study proposes that *NCF4* splice variants generated by functional SNP are important risk factors for mastitis susceptibility in dairy cows.

## Introduction

Mastitis, a prevalent and complex inflammatory disease of the mammary gland, is a consequence of microbial infection and leads to significant economic loss of dairy herds. The innate immune system is the first line of defense against invading pathogens [1]. Nicotinamide dinucleotide phosphate (NADPH) oxidase is an enzymatic complex with a critical role in innate immunity. Phagocyte NADPH oxidase catalyzes the reduction of oxygen to O_2_^−^ and then generates reactive oxygen species (ROS), which are key components of phagocytic microbicidal activity [2]. Studies in animal models and *in vitro* have confirmed the long-standing clinical observation that the NADPH oxidase is critical for defense against catalase-positive bacteria and fungi [3–5]. The enzyme is composed of two membrane-spanning subunits, namely, gp91-phox and p22-phox, which are encoded by CYBB and CYBA, respectively, as well as three cytoplasmic subunits, namely, p40-phox, p47-phox, and p67-phox, which are encoded by NCF4, NCF1, and NCF2, respectively. NCF4, also known as p40phox, is an important gene of the NADPH oxidase complex [6]. NCF4, a key factor in biochemical pathways and innate immune responses, is predominantly expressed in bone marrow cells, including neutrophils, monocytes, basophils, eosinophils, mast cells, megakaryocytes, B cells, and T cells [7,8]. NCF4 specifically interacts with neutrophil cytosolic factor 2 (NCF2/p67-phox) to form a complex with neutrophil cytosolic factor 1 (NCF1/p47-phox), which then interacts with the small G protein RAC1 [9,10]. During bacterial infection, this complex is translocated to the cell membrane of phagocytic cells, where it partners with gp91phox and p22phox to catalyze the production of ROS and facilitate the eradication of invading bacteria [11,12].

Several studies have established that NCF4 is critical for generating superoxides in NCF4-deficient cell lines and gene-targeted mice [11]. In mice lacking NCF4, the PX domain mutant prevented PtdIns(3)P binding, and *in vitro* attack of *Staphylococcus aureus* by neutrophils was reduced to an extent similar to that in the absence of NADPH oxidase activity; moreover, the elimination of *S*. *aureus* was impaired after intraperitoneal injection [13,14]. The decreased levels of NCF4 could diminish Toll-like receptor (TLR) activation and antigen presentation because of inadequate ROS generation [15], thereby prolonging the infection period.

Alternative splicing is a key regulatory mechanism used to generate different mature transcripts from the same primary RNA sequence; the process regulates the eukaryotic expression of immune-related genes and is highly relevant to several diseases, including bovine mastitis [16–19]. Splicing is mediated by spliceosome, which is assembled from snRNAs and protein complexes. The spliceosome is regulated by ubiquitously expressed RNA-binding factors, which interact with *cis*-acting RNA elements to influence spliceosome assembly at nearby splice sites [20]. Spliceosomal recognition and RNA binding factors are involved in mutation-derived and normal alternative mRNA splicing events; however, splicing mutations may alter constitutive splicing, which results in aberrant splicing and clinically abnormal phenotypes [21]. Ma *et al*. [22] revealed the splicing mutation g.8283 C>A in a splice acceptor site of the *PHKG1* intron 9, which resulted in a 32 bp deletion in the open reading frame and generated a premature stop codon. The aberrant transcript expression induces nonsense-mediated decay, which could lead to low protein levels and weak enzymatic activity in affected animals. Our previous study found that SNP c. 1033+2184 C>T in the exonic splicing enhancer (ESE) motif region yields aberrantly spliced *CD46-TV* and is involved in the risk of mastitis caused by *Streptococcus* [23]. An estimated 25% of mutations resumed as missense and nonsense mutations are splicing mutations [24], which can alter the conserved splice sites at exon–intron junctions. However, knowledge remains limited with regard to alternative splicing events and characterization of the *NCF4* splicing mutation, as well as their roles on cattle mastitis susceptibility.

In this study, we hypothesized that the bovine *NCF4* gene may play an important role in bovine mastitis susceptibility, which is regulated by alternative splicing. As such, alternative splicing and splicing-relevant mutation, which can regulate *NCF4* expression, should be studied at the transcriptional level. This study aimed to: (1) investigate whether different splice variants of the *NCF4* gene are present in bovine mammary tissues; (2) analyze the expression patterns of *NCF4* transcripts in healthy and mastitis-infected bovine mammary gland tissues; and (3) explore functional genetic variants associated with cow mastitis susceptibility. Therefore, we attempt to clarify the potential molecular mechanism of *NCF4* expression and its relationship to mastitis caused by pathogenic bacterial infection.

## Materials and Methods

### Ethics statement

All experiments were performed according to the Regulations for the Administration of Affairs Concerning Experimental Animals published by the Ministry of Science and Technology, China in 2004 and approved by the Animal Care and Use Committee from the Dairy Cattle Research Center, Shandong Academy of Agricultural Sciences, Shandong Province, China.

### Sample collection

Mammary tissue samples were collected from the mammary tissues of six normal and six mastitis-infected first-lactating Chinese Holstein cows from a commercial bovine slaughter farm in Jinan, Shandong Province, China. We obtained permission from this slaughterhouse to use the animal parts. Cows were classified based on clinical symptoms and pathogenic bacteria from the milk culture test. The normal cows did not present any clinical symptoms (heat, pain, redness, and swelling of the udder, as well as milk clotting) and was not affected with pathogen infection, as indicated in the culture test. Cows diagnosed with mastitis were first identified based on clinical signs, such as heat, pain, redness, or swelling of the mammary glands, accompanied by the abnormal color or clotting of the milk. The milk isolates of bovine mastitis mammary areas were further tested for pathogenic bacteria by cell culture. The mammary tissues were immediately frozen in liquid nitrogen. Total RNA was extracted from 12 mammary tissues by using RNAsimple Total RNA Kit (Tiangen, Beijing, China) according to the manufacturer's instructions. RNA concentrations were measured with the Biophotometer (Eppendorf). RNA quality was monitored by visualization of ethidium bromide-stained bands in 1% agarose gels after electrophoresis. The samples were stored at −80°C.

A total of 340 Chinese Holstein cows (between 4 and 7 years old, first to fourth parity) from 17 sires were selected from five dairy cattle farms; these farms had complete lactation dairy herd improvement records and were located within the Shandong Agricultural Development Area, P. R. China. Blood samples were collected from the jugular vein with permission from the owners of the animals. Genomic DNA was isolated with the Tianamp Genomic Extraction Kit (Tiangen, Beijing, China). Genomic DNA content was estimated spectrophotometrically and diluted to 50 ng/ml. The DNA samples were stored at −20°C for subsequent analysis. Data on milk SCC, an indicator of mastitis infection, were provided by the Dairy Herd Improvement Laboratory, Dairy Cattle Research Center, Shandong Academy of Agricultural Sciences. The SCC values were transformed into SCS values, which were calculated with the following equation: SCS = log 2 (SCC/100) + 3, where SCC is expressed in cells/μl [25]. The corresponding SCS from dairy herd improvement records were used for association analysis.

### Identification of splice variant

First-strand cDNA was synthesized with the PrimeScript RT Master Mix first-strand cDNA synthesis kit (TaKaRa, Dalian, China). The synthesized cDNA was subjected to PCR with a pair of specific primers NCF4-cDNA (Table 1) to amplify the fragment of the bovine *NCF4* gene (GenBank accession No. NM_001045983.1). DNA bands of PCR products were separated with 1% agarose gel electrophoresis and eluted with the Gel Extraction Kit (BioTeke, Beijing, China). The identification of splice variants was performed according to our previous method [26]. The brief process is as follows. After purification, the PCR products were subcloned into the pEASY-T3 cloning vector (TaKaRa, Dalian, China). The mixture was transformed into *E*. *coli* DH5α. Positive clones were selected and sequenced by BGI Ltd. The sequenced results were compared with the reference *NCF4* mRNA sequence using the DNAMAN v5.2.2 software (LynonBiosoft) for identification of splice variants.

**Table 1:** List of primer sets used in this work.

Primer	Primer *s*equences (5′–3′)	Target	Fragment size (bp)
NCF4- cDNA	F: GAGGCTAGCTGGAGGGAAGT R: CAGCAGGTCTGTCCAACTCA	*NCF4* gene full length cDNA amplification	1228
β-actin gene	F: GCACAATGAAGATCAAGATCATC R: CTAACAGTCCGCCTAGAAGCA	*β-actin* gene qPCR	173
NCF4- reference	F: CACCAACTGGCTACGCTGCTA R: TCTCTGGAACTCCCGCCTCA	Reference transcript qPCR	133
NCF4- TV	F: CTGGCTACGCTGCTATTACTATG R: TCTCTGGAACTCCCGCCTGG	*TV* transcript RT-PCR	175
NCF4- SNP	F: CCAGCTCTGACCCCTTCCAATTC R: CCCCCTCAGCGTCACGGTAG	Genotyping of the SNP g.18174 A>G	178
pSPL3 vector- specific	SD6: TCTGAGTCACCTGGACAACC SA2: ATCTCAGTGGTATTTGTGAGC	Mini-gene expression analysis	263

### Relative expression of bovine NCF4 transcripts

qPCR was conducted to investigate the relative expression of *NCF4* transcripts in the healthy and mastitis-infected bovine mammary gland tissues. The qPCR protocol and calculation of relative expression were previously described by Gao *et al*. [27]. Briefly, qPCR was performed with SYBR green (TaKaRa, Dalian, China) on a Roche LightCycler 480 machine (Roche Applied Science, Mannheim, Germany). The following qPCR profiles were used: 50°C for 2 min; 94°C for 3 min; followed by 40 cycles of 94°C for 30 s, 55°C for 40 s, 68°C for 15 s. The last stage for the dissociation curve analysis was 95°C for 15 s, 60°C for 15 s, and 95°C for 15 s. All qPCR reactions were performed in triplicate. The housekeeping gene *β-actin* was used as reference to normalize data. The template that did not undergo the reverse transcription reaction served as the negative control. Data were analyzed using the 2^-ΔΔCt^ method.

### Analysis and prediction of NCF4 splice variants

The translation of NCF4 splice variant sequence to a protein sequence was performed by ExPASy software (http://web.expasy.org/translate/). The secondary protein structure of bovine *NCF4* splice variants was predicted with SWISS-MODEL (http://swissmodel.expasy.org/). Alterations in the binding site of the splicing factor were predicted by ESEfinder 3.0 (http://rulai.cshl.edu/cgi-bin/tools/ESE3/esefinder.cgi), and alternative splice site prediction was achieved with ASSP (http://wangcomputing.com/assp/index.html).

### Screening and genotyping of splice mutation and association analysis

During the search for alternative splice variants of the *NCF4* gene, we isolated and sequenced *NCF4* cDNA from mammary gland tissue samples and found one SNP g.18174 A>G in the retained 48 bp region of intron 9. To test if the SNP g.18174 A>G was also present in the genomic DNA, we designed the primer pair NCF4-SNP (Table 1) to amplify the region adjacent to intron 9 and exon 10 of the *NCF4* gene. The PCR products from 10 DNA samples were sequenced to verify the authenticity of the SNP.

Direct sequencing of PCR product containing the splicing mutation fragment was used for genotyping of SNP g.18174 A>G. The association between the genotypes and SCS was analyzed using a general least-square model procedure of the SAS statistical analysis software (SAS Institute Inc, Cary, NC, USA). The linear model is expressed as follows: *Y*_*ijkl*_ = μ + *G*_*i*_ + *P*_*j*_ + *E*_*k*_ + *F*_*l*_ + *e*_*ijkl*_, where *Y*_*ijkl*_ is the observed value; μ is the overall mean; *G*_*i*_ is the fixed effect of genotype; *P*_*j*_ is the fixed effect of age; *H*_*k*_ is the effect of farm; *E*_*l*_ is the fixed effect of season; *e*_*ijkl*_ is the random residual error. Values with *P* < 0.05 were considered significant.

### Splicing mini-gene reporter assay

#### Generation of mini-gene constructs

To evaluate the *in vitro* splicing with mini-genes, we amplified a 178 bp genomic fragment spanning the parts of intron 9 and exon 10 of the *NCF4* gene from bovine genomic DNA. After digestion with *Eco*RI and *Xho*I, the segment with the wild-type AA or the mutant type GG of the *NCF4* gene were cloned into the pSPL3 vector (Invitrogen, CA, USA). The clones were transformed into Trans5a cells, plated on agar containing 100 mg/ml ampicillin, and incubated overnight at 37°C. Positive colonies were cultured overnight in lysogeny broth at 37°C. Plasmids were isolated with the Endo-free Plasmid Mini Kit II (Omega, USA). The constructs were directly sequenced to verify the presence of the correct sequences.

#### Cell culture and transfection

HEK 293 T cells were cultured in DMEM medium, with 10% fetal bovine serum (FBS), penicillin (100 U/l), and streptomycin (100 mg/l) at 37°C in 5% CO_2_ atmosphere. For the transfection assay, cells were transferred to six-well culture plates, grown to approximately 80% to 85% confluence and transfected with 4 μg of wild-type or mutant mini-gene constructs with the Lipofectamine 2000 Transfection Reagent (Invitrogen, CA, USA) for 5 h in Opti-MEM medium (Gibco, USA), according to manufacturer’s instructions. As a control, some cells were transfected with pSPL3 lacking the NCF4 insert and some cells were transfected with nothing. All transfections were performed in triplicate and repeated at least thrice in independent experiments.

#### Mini-gene expression analysis

Cells were collected at 24 h post-transfection, and total RNA was extracted with the RNAsimple Total RNA kit (Tiangen, Beijing, China). RNA was reverse transcribed into cDNA. RT-PCR was performed with the PrimerScript RT reagent Kit with gDNA Eraser (TaKaRa, Dalian, China) and the pSPL3 vector specific primers SD6 and SA2 (Table 1). RT-PCR products were separated by electrophoresis on 2% agarose gels, and each DNA band was analyzed by direct sequencing after extraction with a Gel Extraction kit (Omega, USA).

## Results

### Identification of bovine NCF4 splice variants

**Fig 1:**
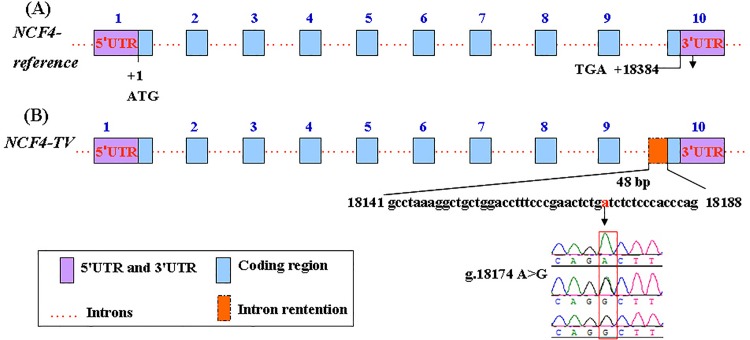
Diagram representation of the alternative splicing pattern of *NCF4* transcripts and results of SNP g.18174 A>G sequencing in the retained 48 bp sequence in intron 9 of the bovine *NCF4* gene. **(A)** Genomic structure of the bovine *NCF4* gene. **(B)** Splicing pattern of the *NCF4*-*TV* splice variant and the sequencing result of SNP g.18174 A>G. The *NCF4*-*TV* transcript retains a 48 bp sequence from intron 9. The position of the A nucleotide in the start codon (ATG) is defined as +1.

cDNA from mammary tissues was used as the template for PCR amplification, which yielded one fragment of approximately 1200 bp. We compared the reference genomic sequence of bovine *NCF4* (GenBank accession number NC_007303.5) and the reference cDNA sequence (GenBank accession number NM_001045983.1). We found two *NCF4* transcripts, which were designated as *NCF4*-*reference* and *NCF4*-*TV*, in mammary gland tissues (Fig 1). The splice variant *NCF4*-*TV* retained a 48 bp fragment of intron 9. The sequence of *NCF4*-*TV* was submitted to the National Center of Biotechnology Information (GenBank accession number KT351731).

**Fig 2:**
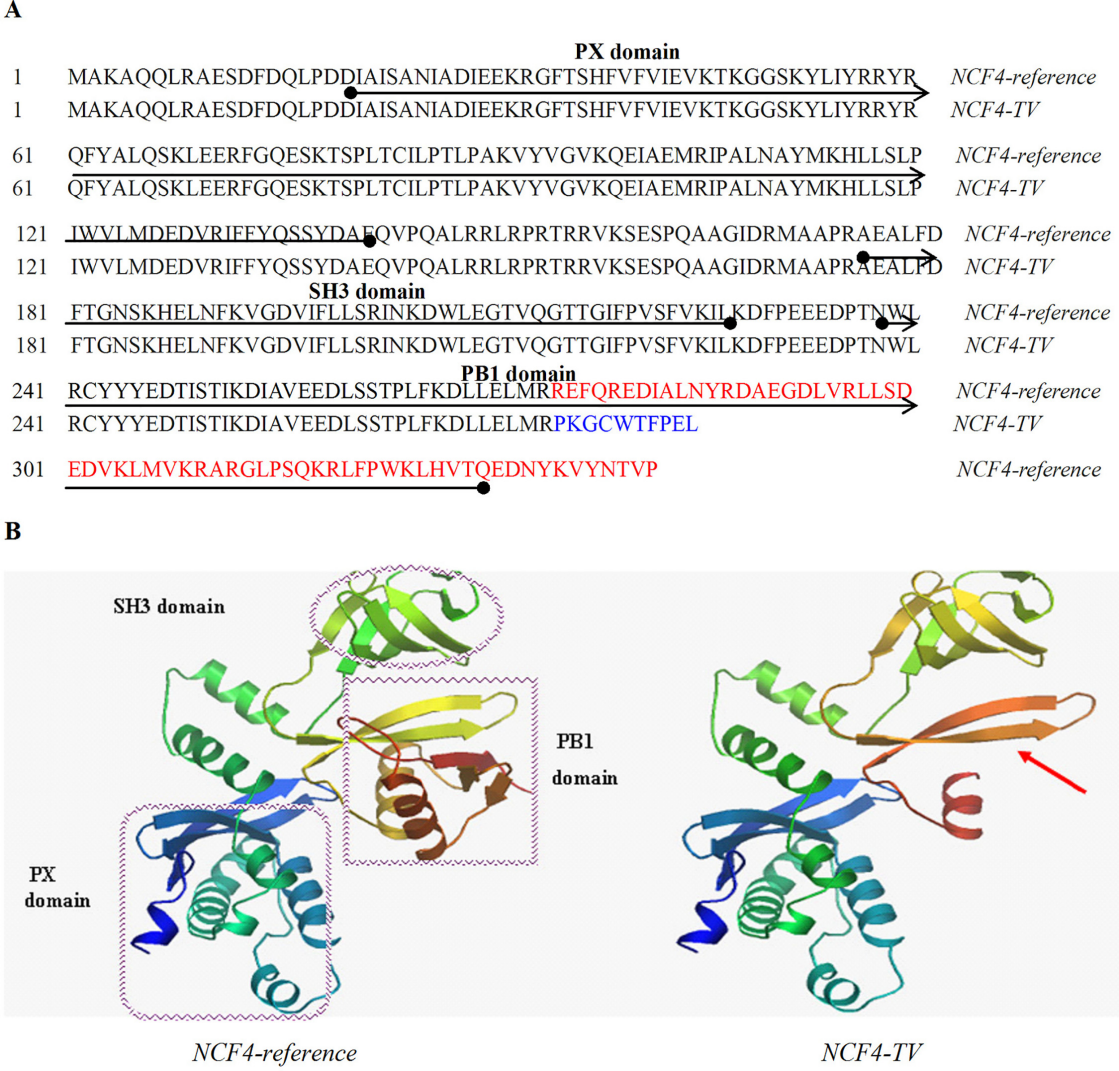
The amino acids sequence, protein structures and domains of the bovine *NCF4*-isoforms. **(A)** The amino acids sequence of bovine *NCF4-reference* and *TV* transcripts. The *NCF4-reference* isoform encoded a 339 aa protein and *NCF4-TV* isoform encoded a 285 aa protein. The amino acids sequence of red and blue marks were differed between the two isoforms. **(B)** The secondary structures and domains of putative isoforms of *NCF4*-*reference* and *NCF4*-*TV*.

The comparison of the bovine *NCF4-reference and NCF4-TV* coding sequences was shown in S1 Fig. We also predicted the amino acids sequence and secondary structure of the putative protein isoforms of *NCF4*-*reference* and *NCF4*-*TV* with ExPASy and SWISS-MODEL, respectively. The amino acids sequence (Fig 2A) and secondary structure differed between the two isoforms (Fig 2B). The *NCF4-reference* isoform encoded a 339 aa protein. Owing to introducing a new stop codon, the putative *NCF4*-*TV* isoform contained 285 aa, of which 64 aa were deleted from the reference sequence and 10 aa were added from retention portion of intron 9. The isoforms shared the same PX and SH3 conserved domains, but *NCF4*-*TV* lost the PB1 domain.

### Relative expression of the NCF4-reference transcript in mammary gland tissues

**Fig 3:**
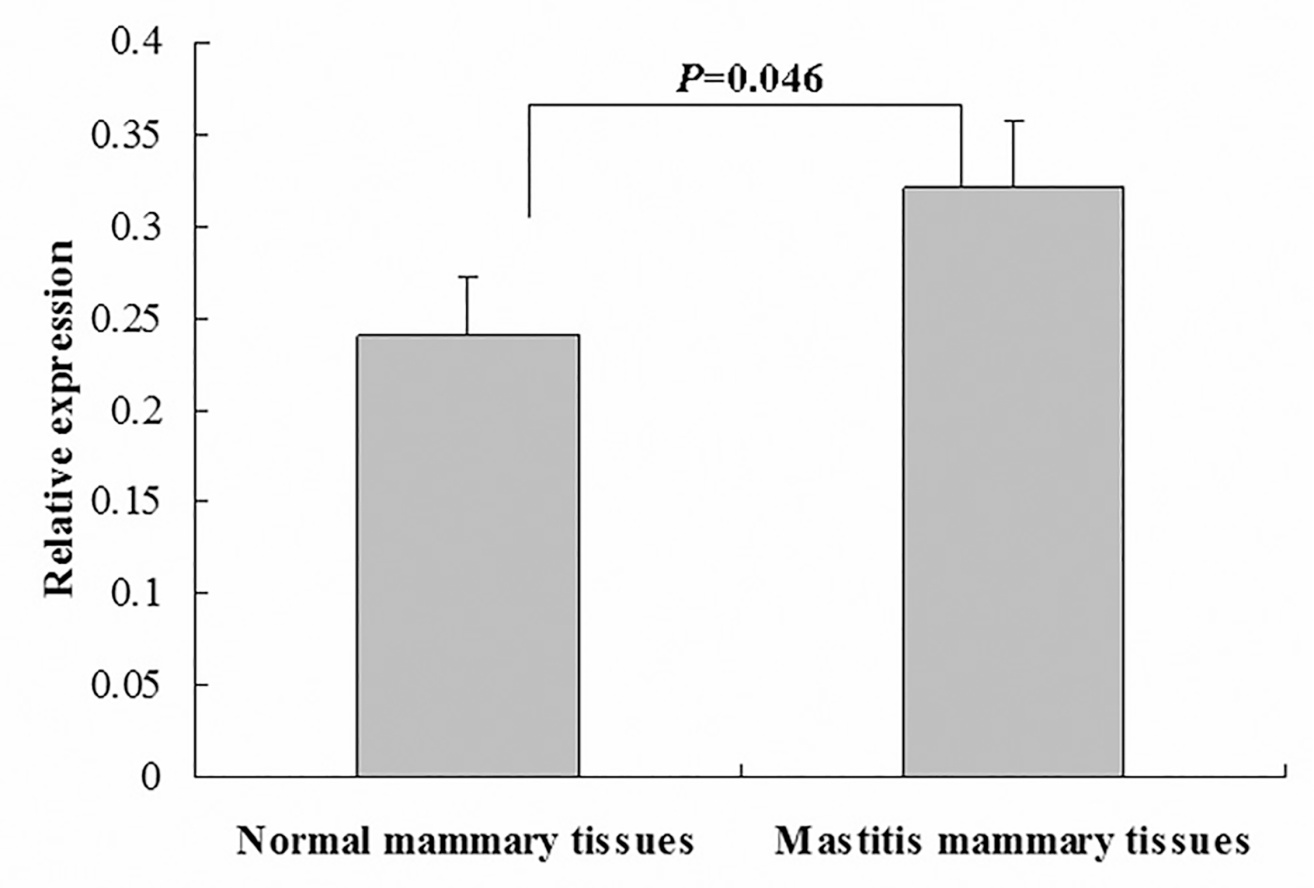
Relative expression of *NCF4-reference* transcripts in normal and mastitic bovine mammary tissues. Gene-specific transcript levels are normalized by the expression of the housekeeping gene *β-actin* in each sample.

Given that fewer *NCF4*-*TV* splice variant clones were found compared with the number of *NCF4-reference* clones, we considered *NCF4-reference* as the main transcript in bovine mammary tissues. Quantitative real-time PCR (qPCR) was performed to determine the relative expression of the bovine *NCF4*-*reference* transcripts in six normal and six mastitic bovine mammary gland tissues (Fig 3). The expression level of *NCF4*-*reference* transcript was relatively higher in mastitic bovine mammary tissues than in the normal tissues (*P* < 0.05).

### Effect of mutation on alternative splice sites

**Fig 4:**
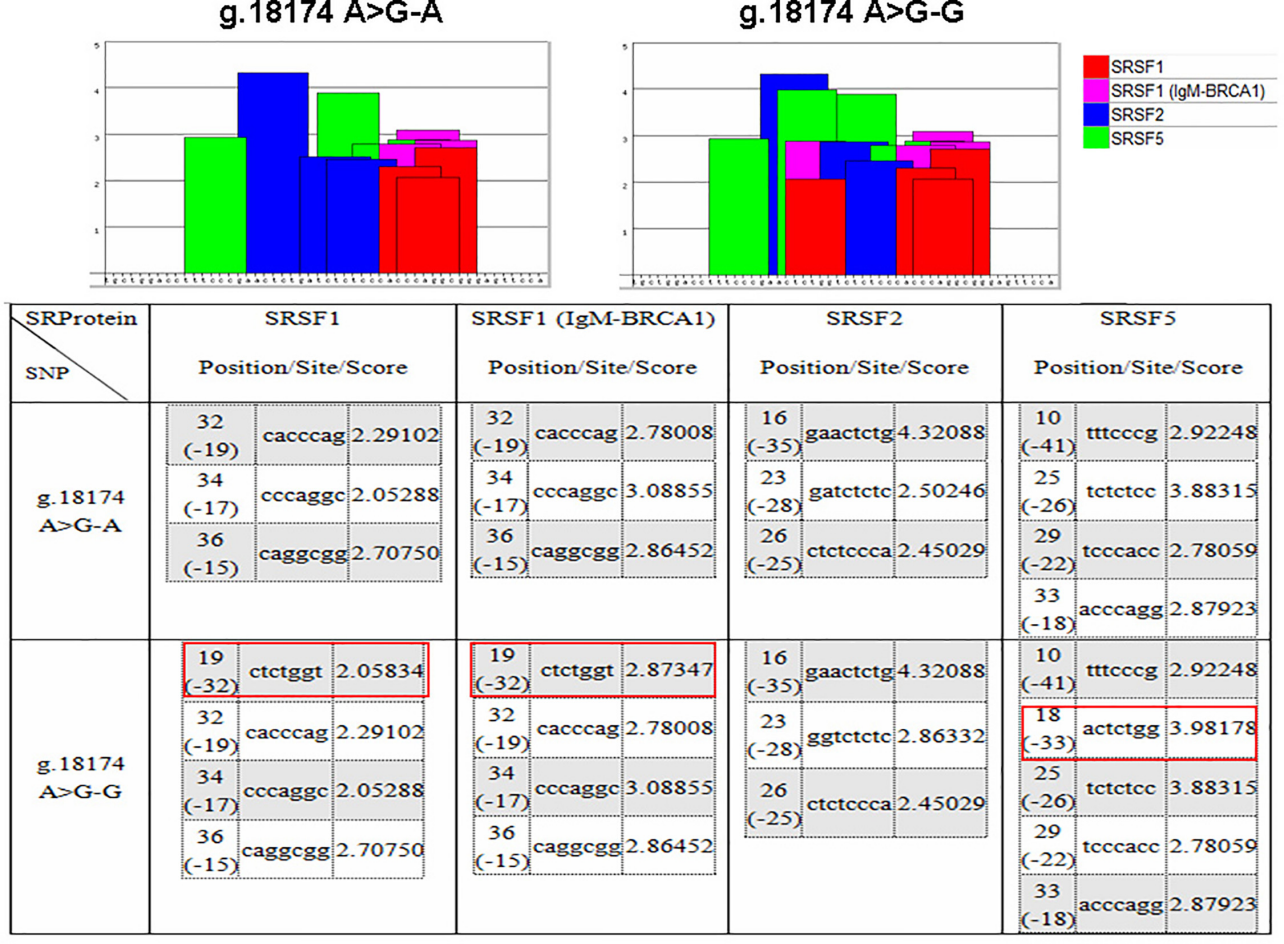
ESE motif threshold scores associated with *NCF4* genotypes. Bar graphs represent scores above the threshold for the ESE motifs in the A or G allele of locus 18174. The red square indicates the introduction of the G allele, relative to the A allele in locus g.18174 A>G, thereby increasing binding sites to the auxiliary splicing proteins: SRSF1, SRSF1 (IgM-BRCA1), and SRSF5. g.18174 A>G-A sequence: 5′ -tgctggacctttcccgaactctgatctctcccacccaggcgggagttcca-3′; g.18174 A>G-G sequence: 5′ -tgctggacctttcccgaactctggtctctcccacccaggcgggagttcca-3′.

To identify the molecular cause of the aberrant *NCF4* splice variant, we sequenced bovine genomic DNA and found a novel SNP g.18174 A>G in the retained 48 bp region of intron 9. The SNP g.18174 A>G was submitted to the National Centre for Biotechnology Information (submitter SNP numbers: ss 1815612795). ESEfinder 3.0 showed that the SNP was located in the ESE motif region. The introduction of allele G, relative to allele A, in the locus g.18174 A>G increased the three binding sites for auxiliary splicing proteins, namely, SRSF1, SRSF1 (IgM-BRCA1), and SRSF5 (Fig 4). The splice site score (8.701) of the mutant sequence was higher than that of the wild-type (8.535) via the Alternative Splice Site Predictor (ASSP) analysis. Therefore, the mutation strengthened the role of the constitutive acceptor splice site, which is located at the 4 bp sequence downstream of the SNP, thereby modifying the *NCF4*-*TV* splicing of mature mRNA during *NCF4* transcription.

### Mini-gene splicing assay

**Fig 5:**
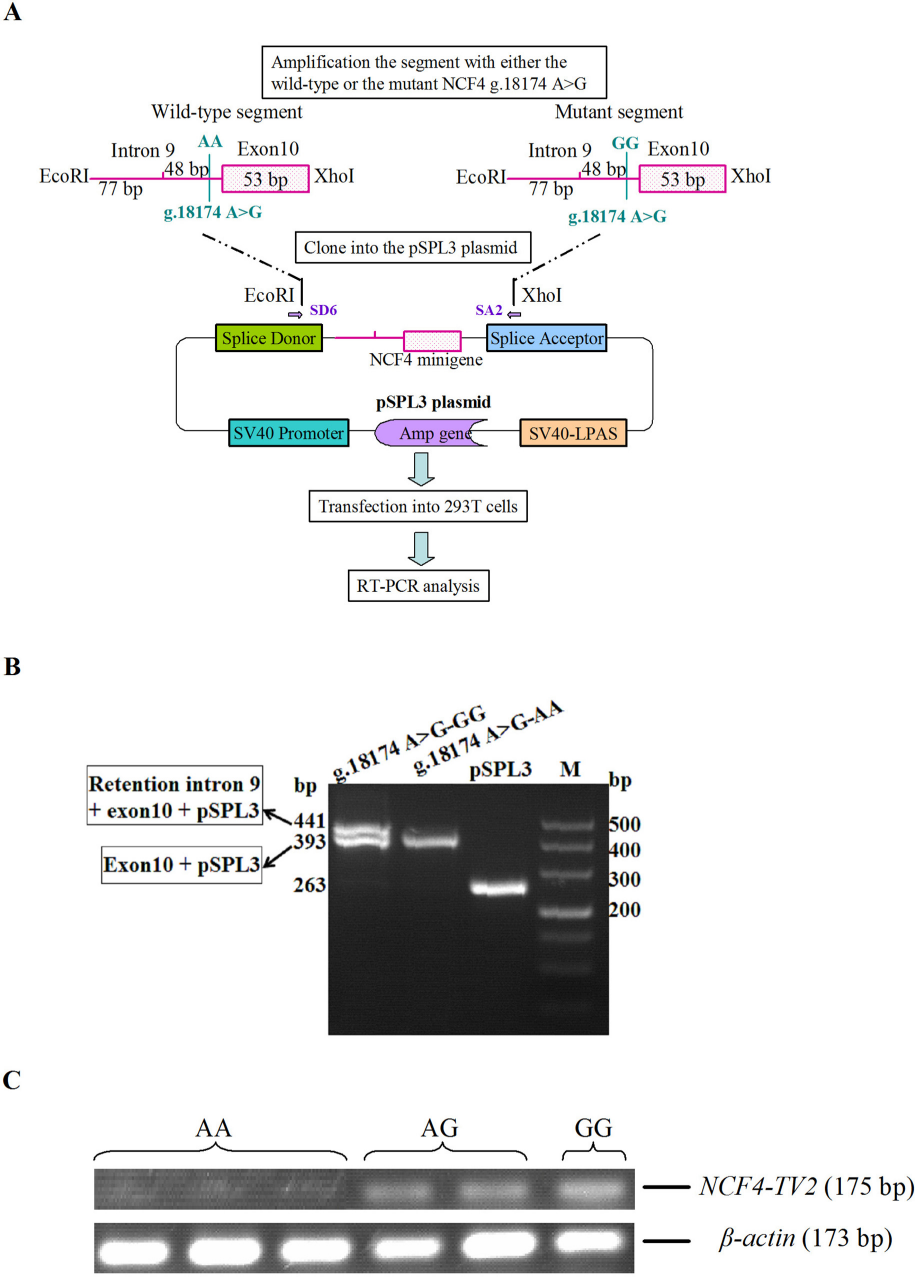
SNP g.18174 A>G induces aberrant *NCF4*-*TV* splicing. **(A)** Schematic representation of the *NCF4* mini-genes used in the functional splicing assay. The wild-type and mutant fragments contained 125 bp of intron 9 and 53 bp of exon 10; fragments harboring the A or G allele were separately cloned into the *Eco*RI and *Xho*I cloning sites of the pSPL3 vector. Two mini-gene expression vectors were transiently transfected into 293T cells. **(B)** RT-PCR analysis of the *NCF4* spliced transcripts on a 2% agarose gel. RT-PCR products were amplified from the total RNA of 293T cells transfected with the wild-type and mutant (g.18174 A>G) *NCF4* mini-gene constructs. The size of the RT-PCR product (441 bp) corresponded to the amplified portion of intron 9 (77 bp), the retained portion of intron 9 (48 bp), the amplification of exon 10 (53 bp), and the pSPL3 control plasmid (263 bp). The size of the RT-PCR product (393 bp) corresponded to the amplified portion of intron 9 (77 bp), the amplification of exon 10 (53 bp), and the pSPL3 control plasmid (263 bp). **(C)** Electrophoresis of RT-PCR products showing the presence and abundance of *NCF4*-*TV* transcript in bovine mammary samples with three *NCF4* SNP g.18174 A>G genotypes. Expression of the *NCF4*-*TV* transcript is highest in mammary samples from GG animals, followed by those from AG and AA individuals.

To test whether the retention of the 48 bp *NCF4*-*TV* splice variant in intron 9 was caused by the internal mutation g.18174 A>G, we assessed the effect of the variant on splicing through the mini-gene splicing assay. Two mini-gene expression vectors, which carried either the wild-type AA or the mutant type GG fragment of *NCF4*, were constructed and transiently transfected into 293T cells (Fig 5A). The mini-gene transcripts in the transfected cells were analyzed by RT-PCR with the pSPL3 vector-specific primers SD6 and SA2. We found two amplicons of 393 and 441 bp from the mutant GG construct and only one amplicon of 393 bp from the wild-type AA construct (Fig 5B). Sequencing analysis revealed that the truncated amplicon (393 bp) corresponded to the portions of intron 9 (77 bp) and exon 10 (53 bp) of bovine *NCF4* and the empty pSPL3 control (263 bp) sequence, respectively. The second amplicon (441 bp) included a portion of intron 9 (77 bp), retained portion of intron 9 (48 bp), and a portion of exon 10 (53 bp) of *NCF4*, as well as the empty pSPL3 control (263 bp) sequence. The mini-gene splicing assay demonstrated that SNP g.18174 A>G is responsible for the *NCF4*-*TV* aberrant splicing, which produces the 48 bp retained portion in intron 9.

### Effect of SNP g.18174 A>G on NCF4 mRNA levels

We genotyped six Chinese Holstein cows, namely, 3 AA, 2 AG, and 1 GG individuals. RT-PCR analysis with the NCF4-TV primer pair revealed that the *NCF4*-*TV* transcript was expressed in the GG individual compared with the AA individuals (Fig 5C). Furthermore, we performed a quantification analysis on the expression of *NCF4*-*TV* transcript by Image J software. The results suggest that the *NCF4*-*TV* expression is up-regulated by 1.62- and 6-fold in the cows with the genotype GG compared with individuals with the genotypes AG and AA, respectively. However, larger sample sets are still needed to confirm the expression quantification.

### Association between the SNP g.18174 A>G and milk SCS in Chinese Holstein cows

To investigate whether SNP g.18174 A>G was associated with mastitis in dairy cows, we evaluated the effects of this SNP on milk SCS values in 340 Chinese Holstein cows. We first amplified the PCR product containing the splicing mutation fragment and then genotyped each individual by direct sequencing. The frequencies of the AA, AG, and GG genotypes were 48.24%, 36.76%, and 15.00%, respectively. Subsequently, we analyzed the relationship between the three genotypes and milk SCS. The individuals with the AA genotype presented lower SCS values than those with the GG genotype (*P* < 0.05, Table 2).

**Table 2:** Least squares mean and standard error of SCS in different genotypes of the *NCF4* SNP g.18174 A>G in Chinese Holstein cows.

Genotype	Sample number	Genotypic frequencies (%)	Allelic frequencies (allele) (%)	SCS
AA	164	48.24	66.62(A)	4.26 ± 0.27^b^
AG	125	36.76		4.51 ± 0.42^a^ ^b^
GG	51	15.00	33.38(G)	4.90 ± 0.29^a^

^a,b^ Means with different superscripts are significantly different (*P* < 0.05).

## Discussion

This study utilized the combined RT-PCR, clone sequencing, mini-gene splicing system *in vitro*, and association analysis to characterize a novel *NCF4* transcript in mammary gland tissues. We found an SNP in intron 9, which could cause aberrant intron retention. This SNP produced the splice variant *NCF4-TV*, which is associated with mastitis susceptibility in cows.

The p40phox protein encoded by *NCF4* is a regulatory component of the superoxide-producing phagocyte NADPH oxidase, an important multicomponent enzyme system for host defense [28]. NADPH oxidase generates ROS by catalyzing the one-electron reduction of oxygen into the superoxide anion radical [29]. This multi-subunit complex plays a central role in the defense mechanisms against invading microbes. Mastitis is an inflammatory disease caused by various pathogenic microorganisms in dairy cattle. NCF4 is an essential innate immunity gene with an important role in bovine mastitis.

The human *NCF4* gene shows a pattern of diversity [1], with four splice variants deposited in the Ensembl database (www.ensembl.org). The four different human transcripts encoded four types of protein with sizes of 339 aa, 348 aa, 212 aa, and 166 aa. In the present study, a novel *NCF4* transcript variant was identified in the mammary gland tissues of Chinese Holsteins. The multiple mRNA transcripts produced by alternative splicing from a single primary RNA may exhibit diverse functional properties [30,31]. SWISS-MODEL predicted that the putative *NCF4-TV* isoform demonstrated different protein secondary structures, without a functional domain, because of intron retention. Two *NCF4* isoforms share the common PX domain, which is important for the basic immunity-associated functions of the NCF4 protein. But due to lack of specific bovine NCF4 antibody, a western blot analysis had not been done to detect the presence of both protein variants. This requires us to further study. The novel putative *NCF4-TV* isoform would influence several functions because of the lack of the PB1 domain, which is required for the formation of a complex between the p40phox and p67phox subunits to maximize the activation of NADPH oxidase [32]. The NADPH oxidase activity regulates phagosomal pH, and the derivative ROS can function as signaling molecules within and between neighboring immune cells [33]. Meanwhile, reduced ROS levels in these cells may influence antigen processing, immunoregulation, control of cell activation, and differentiation [34]. Deletion of the PB1 domain would affect the interaction between NCF4 and NCF2, which is an important risk factor for increased systemic lupus erythematosus, a typical systemic autoimmune disease [35]. Subsequently, qPCR analysis showed that the expression of the main transcript *NCF4-reference* were up-regulated in mastitic bovine mammary tissues relative to normal tissues. These results imply that the *NCF4* gene is a risk factor for mastitis susceptibility by altering gene expression at the transcription level by alternative splicing.

Splicing regulation involves multiple splicing factors, including the SR and hnRNP protein families. Sequence variants, such as SNPs, affect protein-binding sites (or mutations in the binding proteins themselves) and contribute to aberrant splicing [36]. In the present study, we found the SNP g.18174 A>G in the retained portion of intron 9 of the *NCF4*-*TV* splice variant. First, we used the ESEfinder software to predict whether the SNP would affect binding capacity with splicing proteins. The prediction results showed that relative to the A allele, the G allele increased the number of binding sites to the auxiliary splicing proteins: SRSF1, SRSF1 (IgM-BRCA1), and SRSF5. An intronic mutation (c.903+469T>C) in the *MTRR* gene, creates an SRSF1 binding motif, which leads to intron retention [37]. SR proteins (serine/arginine-rich splicing factor, SRSF) are a family of structurally related and highly conserved cellular splicing factors and primarily modulate alternative splicing. Therefore, the increased binding of the three splicing factors may generate the *NCF4*-*TV* splice variant.

To analyze further the contribution of SNP g.18174 A>G in the regulation of *NCF4*-*TV*, we constructed two mini-gene expression vectors, which were transiently transfected into 293T cells and analyzed by RT-PCR. In contrast to the wild-type AA construct, the mutant GG construct permitted the retention of the 48 bp intron 9 sequence. This experiment was consistent with the predicted results. The SNP g.18174 A>G contributed to the aberrant splicing of *NCF4*-*TV*. The expression of *NCF4*-*TV* transcripts in GG individuals was higher than in AA individuals, thereby further confirming our view.

Mutations that affect alternative splice variants account for at least 15% of disease-related mutations [36]. The associations reported in GWAS between SNP rs4821544 in human *NCF4* and Crohn’s disease confirmed the involvement of NADPH genes in the pathogenesis of common inflammatory-related diseases [38,39]. Zhang *et al*. [19] reported that the SNP g.10766 T>C caused the production of the aberrant splice variant *NCF1-TV1* and associated with increased milk somatic cell score in cows. To test whether the identified g.18174 A>G SNP has an important role in bovine mastitis, we performed association analysis between the three genotypes and SCS in Chinese Holsteins. The individuals with AA genotypes exhibited lower SCS values than those with the GG genotypes. During mastitis infection, the recruitment of neutrophils into the mammary gland increases somatic cell count (SCC), which is an indirect selection tool for reduced mastitis [40]. The SNP g.18174 A>G of *NCF4* may contribute to genome-assisted selection of SNP panels to improve mastitis resistance traits on a breed basis.

## Supporting Information

S1 Fig Comparisons of bovine *NCF4-reference* and *NCF4-TV* CDS regions. Different sequences within the two transcripts are indicated in red and blue.
(PDF)
