# Supplementary material for: Role of an SNP in Alternative Splicing of Bovine NCF4 and Mastitis Susceptibility
Source: PLoS One. 2015 Nov 24;10(11):e0143705. doi: 10.1371/journal.pone.0143705 (PMC4658021; doi:10.1371/journal.pone.0143705)
Supplement: S1 Fig — Different sequences within the two transcripts are indicated in red and blue. (PDF) [file pone.0143705.s001.pdf]

Supplementary data

Figure S1 Comparisons of bovine *NCF4-reference* and *NCF4-TV* CDS regions. Different sequences within the two transcripts are indicated in italic letters.

```
                                                                         NCF4
1     ATGGCCAAGGCACAGCAACTTCGGGCTGAGAGCGACTTCGACCAGCTTCCTGACGACATT      -reference
      ||||||||||||||||||||||||||||||||||||||||||||||||||||||||||||
1     ATGGCCAAGGCACAGCAACTTCGGGCTGAGAGCGACTTCGACCAGCTTCCTGACGACATT      -TV

61    GCCATCTCAGCCAACATCGCTGACATTGAGGAGAAAAGAGGCTTCACCAGCCACTTTGTT     -reference
      ||||||||||||||||||||||||||||||||||||||||||||||||||||||||||||
61    GCCATCTCAGCCAACATCGCTGACATTGAGGAGAAAAGAGGCTTCACCAGCCACTTTGTT     -TV

121   TTTGTCATCGAGGTAAAGACGAAAGGGGGGTCCAAGTACCTCATCTACCGCCGCTACCGC     -reference
      ||||||||||||||||||||||||||||||||||||||||||||||||||||||||||||
121   TTTGTCATCGAGGTAAAGACGAAAGGGGGGTCCAAGTACCTCATCTACCGCCGCTACCGC     -TV

181   CAGTTCTACGCCTTACAGAGCAAGCTGGAGGAGCGCTTCGGTCAGGAGAGCAAGACCAGC     -reference
      ||||||||||||||||||||||||||||||||||||||||||||||||||||||||||||
181   CAGTTCTACGCCTTACAGAGCAAGCTGGAGGAGCGCTTCGGTCAGGAGAGCAAGACCAGC     -TV

241   CCCTTAACCTGTATCCTCCCCACGCTCCCAGCCAAAGTCTACGTGGGTGTGAAACAGGAG     -reference
      ||||||||||||||||||||||||||||||||||||||||||||||||||||||||||||
241   CCCTTAACCTGTATCCTCCCCACGCTCCCAGCCAAAGTCTACGTGGGTGTGAAACAGGAG     -TV

301   ATTGCCGAGATGCGAATACCTGCCCTCAACGCCTACATGAAGCACCTCCTCAGCCTGCCC     -reference
      ||||||||||||||||||||||||||||||||||||||||||||||||||||||||||||
301   ATTGCCGAGATGCGAATACCTGCCCTCAACGCCTACATGAAGCACCTCCTCAGCCTGCCC     -TV

361   ATCTGGGTGCTGATGGACGAGGACGTTCGCATCTTCTTCTACCAGTCGTCCTACGACGCC     -reference
      ||||||||||||||||||||||||||||||||||||||||||||||||||||||||||||
361   ATCTGGGTGCTGATGGACGAGGACGTTCGCATCTTCTTCTACCAGTCGTCCTACGACGCC     -TV

421   GAGCAGGTGCCTCAAGCGCTCCGGCGGCTCCGCCCGCGCACCCGGCGAGTAAAAAGCGAG     -reference
      ||||||||||||||||||||||||||||||||||||||||||||||||||||||||||||
421   GAGCAGGTGCCTCAAGCGCTCCGGCGGCTCCGCCCGCGCACCCGGCGAGTAAAAAGCGAG     -TV

481   TCCCCACAAGCTGCTGGCATTGACCGCATGGCAGCTCCACGAGCAGAGGCCCTGTTTGAT     -reference
      ||||||||||||||||||||||||||||||||||||||||||||||||||||||||||||
481   TCCCCACAAGCTGCTGGCATTGACCGCATGGCAGCTCCACGAGCAGAGGCCCTGTTTGAT     -TV

541   TTCACTGGGAACAGCAAACATGAGCTGAATTTCAAAGTTGGAGATGTGATCTTCCTTCTC     -reference
```

| | | | | | | | | | | | | | | | | | | | | | | | | | | | | | | | | | | | | | | | | | | | | | | | | | | | | | | | | | | |

541 TTCACTGGGAACAGCAAACATGAGCTGAATTTCAAAGTTGGAGATGTGATCTTCCTTCTC *-TV*

601 AGTCGGATCAATAAAGACTGGCTGGAGGGCACTGTCCAGGGAACCACAGGCATCTTCCCA *-reference*

| | | | | | | | | | | | | | | | | | | | | | | | | | | | | | | | | | | | | | | | | | | | | | | | | | | | | | | | | | | |

601 AGTCGGATCAATAAAGACTGGCTGGAGGGCACTGTCCAGGGAACCACAGGCATCTTCCCA *-TV*

661 GTGTCCTTTGTGAAGATCCTCAAGGACTTCCCAGAGGAGGAAGACCCCACCAACTGGCTA *-reference*

| | | | | | | | | | | | | | | | | | | | | | | | | | | | | | | | | | | | | | | | | | | | | | | | | | | | | | | | | | | |

661 GTGTCCTTTGTGAAGATCCTCAAGGACTTCCCAGAGGAGGAAGACCCCACCAACTGGCTA *-TV*

721 CGCTGCTATTACTATGAGGACACCATCAGCACCATCAAGGACATTGCAGTGGAGGAGGAC *-reference*

| | | | | | | | | | | | | | | | | | | | | | | | | | | | | | | | | | | | | | | | | | | | | | | | | | | | | | | | | | | |

721 CGCTGCTATTACTATGAGGACACCATCAGCACCATCAAGGACATTGCAGTGGAGGAGGAC *-TV*

781 CTCAGCAGCACCCCACTCTTCAAGGACTTGCTGGAGCTCATGAG................................ *-reference*

| | | | | | | | | | | | | | | | | | | | | | | | | | | | | | | | | | | | | | | | | | | |

781 CTCAGCAGCACCCCACTCTTCAAGGACTTGCTGGAGCTCATGAG*GCCTAAAGGCTGCTGG* *-TV*

825 .... *GCGGGAGTTCCAGAGAGAGGACATCGCCCTCAACTACCGTGAC* *-reference*

841 *ACCTTTCCCGAACTC**TGA*** *-TV*

868 *GCTGAGGGGGACCTGGTTCGGCTGCTGTCAGATGAGGACGTGAAGCTCATGGTGAAGCGG* *-reference*

928 *GCCCGAGGTCTCCCCTCCCAGAAGCGTCTCTTCCCCTGGAAGCTGCACGTCACCCAGGAG* *-reference*

988 *GACAACTACAAGGTCTACAACACAGTCCCC**TGA*** *-reference*
